# Effects of aging on otolith morphology and functions in mice

**DOI:** 10.3389/fnins.2024.1466514

**Published:** 2024-10-16

**Authors:** Keita Ueda, Takao Imai, Taeko Ito, Tadao Okayasu, Shotaro Harada, Takefumi Kamakura, Kazuya Ono, Tatsuya Katsuno, Tatsuhide Tanaka, Kouko Tatsumi, Hiroshi Hibino, Akio Wanaka, Tadashi Kitahara

**Affiliations:** ^1^Department of Otolaryngology-Head and Neck Surgery, Nara Medical University, Nara, Japan; ^2^Department of Otorhinolaryngology-Head and Neck Surgery, Osaka University Graduate School of Medicine, Osaka, Japan; ^3^Division of Glocal Pharmacology, Department of Pharmacology, Graduate School of Medicine, Osaka University, Osaka, Japan; ^4^Electron Microscopy Facility, Center for Anatomical Studies, Graduate School of Medicine, Kyoto University, Kyoto, Japan; ^5^Department of Anatomy and Neuroscience, Faculty of Medicine, Nara Medical University, Nara, Japan; ^6^AMED-CREST, AMED, Osaka, Japan

**Keywords:** otolith, otoconia, micro-computed tomography, linear vestibulo-ocular reflex, mice

## Abstract

**Background:**

Increased fall risk caused by vestibular system impairment is a significant problem associated with aging. A vestibule is composed of linear acceleration-sensing otoliths and rotation-sensing semicircular canals. Otoliths, composed of utricle and saccule, detect linear accelerations. Otolithic organs partially play a role in falls due to aging. Aging possibly changes the morphology and functions of otoliths. However, the specific associations between aging and otolith changes remain unknown. Therefore, this study aimed to clarify these associations in mice.

**Methods:**

Young C56BL/6 N (8 week old) and old (108–117 weeks old) mice were used in a micro-computed tomography (μCT) experiment for morphological analysis and a linear acceleration experiment for functional analysis. Young C56BL/6 N (8 week old) and middle-aged (50 week old) mice were used in electron microscopy experiments for morphological analysis.

**Results:**

μCT revealed no significant differences in the otolith volume (*p* = 0.11) but significant differences in the otolith density (*p* = 0.001) between young and old mice. μCT and electron microscopy revealed significant differences in the structure of striola at the center of the otolith (μCT; *p* = 0.029, electron microscopy; *p* = 0.017). Significant differences were also observed in the amplitude of the eye movement during the vestibulo-ocular reflex induced by linear acceleration (maximum amplitude of stimulation = 1.3G [*p* = 0.014]; maximum amplitude of stimulation = 0.7G [*p* = 0.015]), indicating that the otolith function was worse in old mice than in young mice.

**Discussion:**

This study demonstrated the decline in otolith function with age caused by age-related morphological changes. Specifically, when otolith density decreased, inertial force acting on the hair cells decreased, and when the structure of striola collapsed, the function of cross-striolar inhibition decreased, thereby causing a decline in the overall otolith function.

## 1 Introduction

Increased fall risk is a significant problem associated with aging (Overstall et al., 1977; Karlsen et al., 1981; Maki et al., 1994; Baloh et al., 1998). The deterioration in dynamic balance causes falls because of impairment of the vestibular system (Karlsen et al., 1981; DiZio and Lackner, 1990; Baloh et al., 1993), muscles, tendons, joint proprioceptors (Whipple et al., 1987; Alexander, 1994; Tinetti et al., 1995), and visual coordination (Gerson et al., 1989; Manchester et al., 1989; Lord and Ward, 1994). The vestibular system comprises of otoliths and semicircular canals. Otoliths, composed of utricle and saccule, detect linear accelerations and semicircular canals, detect rotational acceleration (Ramos de Miguel et al., 2020; Tadokoro et al., 2024).

There are several reports on age-related decline of otoliths and semicircular canals, including function and organization. Ross et al. (1976) examined human otoliths using electron microscopy and showed that otoconia in older people were degenerated and split compared with otoliths in younger people. In humans, the ocular vestibular evokes myogenic potential, which is thought to reflect utricle function, and shows a decrease in amplitude and an increase in latency with age (Iwasaki et al., 2009; Maheu et al., 2015). A previous study suggested an overall decline in semicircular canal as well as otolith function associated with aging in human (Agrawal et al., 2012). A different study showed that otolith dysfunction occurs in FBV/N mice, but later than hearing loss (Wan et al., 2019). Moreover, age-related reduction of hair cells was suggested to be less common in the otoliths and more common in the semicircular canals in Fisher 344 rats (Nakayama et al., 1994). The blood flow in the oval sac is reduced with age, which may affect otolith function in Fisher 344 rats (Lyon and Davis, 2002). In humans, when otoconia peel off from otoliths and stray into the semicircular canal, they cause recurrent rotational vertigo with each head position change, rendering affected people more prone to falls (Bhattacharyya et al., 2017).

Otoliths appear to be partially responsible for age-related falls (Semenov et al., 2016). Aging changes the otolith morphology and decreases their function; however, the specific relationship between aging and these changes is not entirely clear, thus further research is warranted. Here, we aimed to clarify these relationships in mice. We evaluated the otolith morphology via micro-computed tomography (μCT) and electron microscopy. Additionally, we analyzed the otolith functions. We previously reported that the otoliths in mice were stimulated and induced eye movements, known as the linear vestibulo-ocular reflex (LVOR), during linear translation (Harada et al., 2021). Analysis of eye movements induced by LVOR can aid in assessing otolith function because eye movement becomes weak when the otoliths are damaged. By performing these experiments on mice of different ages and comparing the results, we can examine the changes in morphology and function of the otoliths as they age. The results of this study will be relevant for future research on otoconia.

## 2 Materials and methods

All procedures were performed according to the Ethics Committee for Animal Experiments guidelines of Nara Medical University (Nara, Japan). Young and old C56BL/6 N mice were used in this study.

### 2.1 Animals

For the μCT experiment, five male and five female 8-week-old and five male and five female 117-week-old mice were used. One 117-week-old male mouse died during the experiment, thus 19 mice (*n* = 38 ears) were included in this study. The weights of the 8- and 117-week-old mice were 18–21 and 34–40 g, respectively. The 8-week-old group was bred in the Animal Experimentation Building of Nara Medical University, and the 117-week-old group was purchased from CLEA Japan (Tokyo, Japan) and maintained in the Animal Experimentation Building of Nara Medical University.

For the electron microscopy experiment, 8-week-old (*n* = 5) and 50-week-old (*n* = 5) male mice were used.

For the LVOR experiment, we used 10 male and 10 female 8-week-old mice and 10 male and 10 female 108-week-old mice. Therefore, a total of 40 mice were used for this experiment. The 8-week-old mice weighed 16–18 g, and 108-week-old mice weighed 27–34 g. The mice were purchased from CLEA Japan and kept for 1 week at the animal experimental facility of Osaka University to acclimatize them to the environment before the experiment.

### 2.2 Morphological changes in otoliths due to aging

#### 2.2.1 Three-dimensional μCT

##### 2.2.1.1 Morphological analysis of otoliths via μCT imaging

The inner ears of mice were scanned using the Cosmosan FX μCT system (Summit Pharmaceuticals International, Tokyo, Japan). As mouse otoliths range in size from 0.1 to 25 μm (Honda et al., 2015), imaging was performed to capture the finest detail with this system (200 μA, 30 kV, 2 min, FOV 10 μm). To prevent any errors in CT images caused by mouse body movements, a three-drug anesthetic mixture of 1.875 mL medetomidine hydrochloride (1.0 mg/mL; Nippon Zenyaku Kogyo), 2.0 mL mitazolam (5.0 mg/mL; Astellas Pharma Inc.), and 2.5 mL butorphanol tartrate (5.0 mg/mL; Meiji Seika Kaisha) was diluted in 18.6 mL distilled water and intraperitoneally injected into the mice at 0.01 mL/g of body weight. After 15 min, their backs were grasped with tweezers to confirm that the animals were sufficiently sedated without body movement. The mice were placed in an animal holder (KN-325-A, rat holder; Natsume Seisakusho Co., Tokyo, Japan). The animal holder allowed us to obtain CT images with almost the same head angle for all mice.

Attractive software (PixSpace, Ltd., Fukuoka, Japan) was used for image analysis. This is a reliable software used in many studies (Inui et al., 2016; Ito et al., 2019; Yoshida et al., 2019; Takasu et al., 2020; Kiuchi et al., 2021; Kohada et al., 2022).

**Figure 1:**
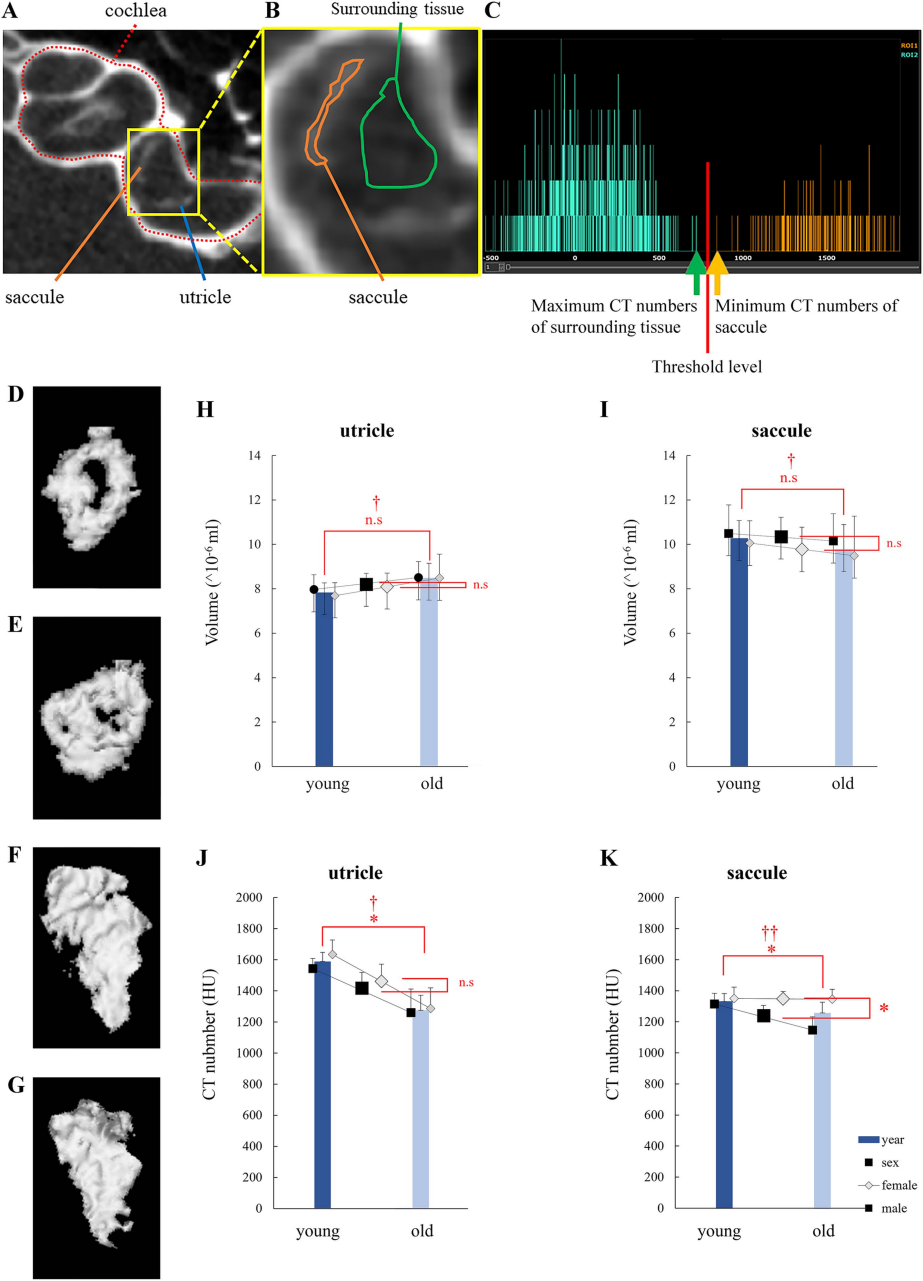
Otolith analysis via micro-computed tomography (μCT). **(A)** μCT image of the left inner ear of a mouse showing the bony labyrinth, cochlea, utricle, and saccule. **(B)** An enlarged view of the otolith organ is shown in **(A)**. Central: Saccule is surrounded by an orange-framed region of interest (ROI), and a green-framed ROI surrounds the surrounding tissue. The surrounding tissue was filled with endolymph or perilymph. **(C)** CT histogram of ROIs. CT numbers in the saccule were distributed more than those in the surrounding tissue. **(D)** 3D model of a young mouse utricle. The low-density area at the center indicates the striola. **(E)** 3D model of an old mouse utricle. The low-density area is smaller than that observed in **(D)**. **(F)** 3D model of a young mouse saccule. Striola cannot be identified. **(G)** 3D model of an old mouse saccule. Striola cannot be identified. **(H)** Utricle volume. No significant differences were observed in age and sex. **(I)** Saccule volume. No significant differences were observed in age and sex. **(J)** CT numbers in the utricle. Significant differences were observed in age and sex. No significant interactions were observed (age, *p* = 0.001; sex, *p* = 0.32; interaction, *p* = 0.58; *n* = 38). **(K)** CT numbers in the saccule. Significant differences were observed in age but not in sex. An interaction effect was also observed (age, *p* = 0.026; sex, *p* = 0.003; interaction, *p* = 0.034; *n* = 38). *indicates *p* < 0.05. †indicates no interaction. ††indicates interaction.

In the μCT images, otolith spots were observed (Figure 1A). The region of interest (ROI) was set by manually specifying the region containing the otolith spot without including the temporal bone (Figure 1B). The CT number in the ROI of otolith (utricle and saccule; *α*; Figures 1B,C, orange line) was more significant than that in the ROI of the surrounding tissue (*β*; Figures 1B,C, green line). As shown in Figure 1C, the otolith region was detected as the area with a high CT number (α + β)/2 using the Attractive image analysis software. Attractive also constructed the three-dimen3sional otolith model (Figures 1D–G).

###### 2.2.1.1.1 Comparison of tissue slide and μCT images

**Figure 2:**
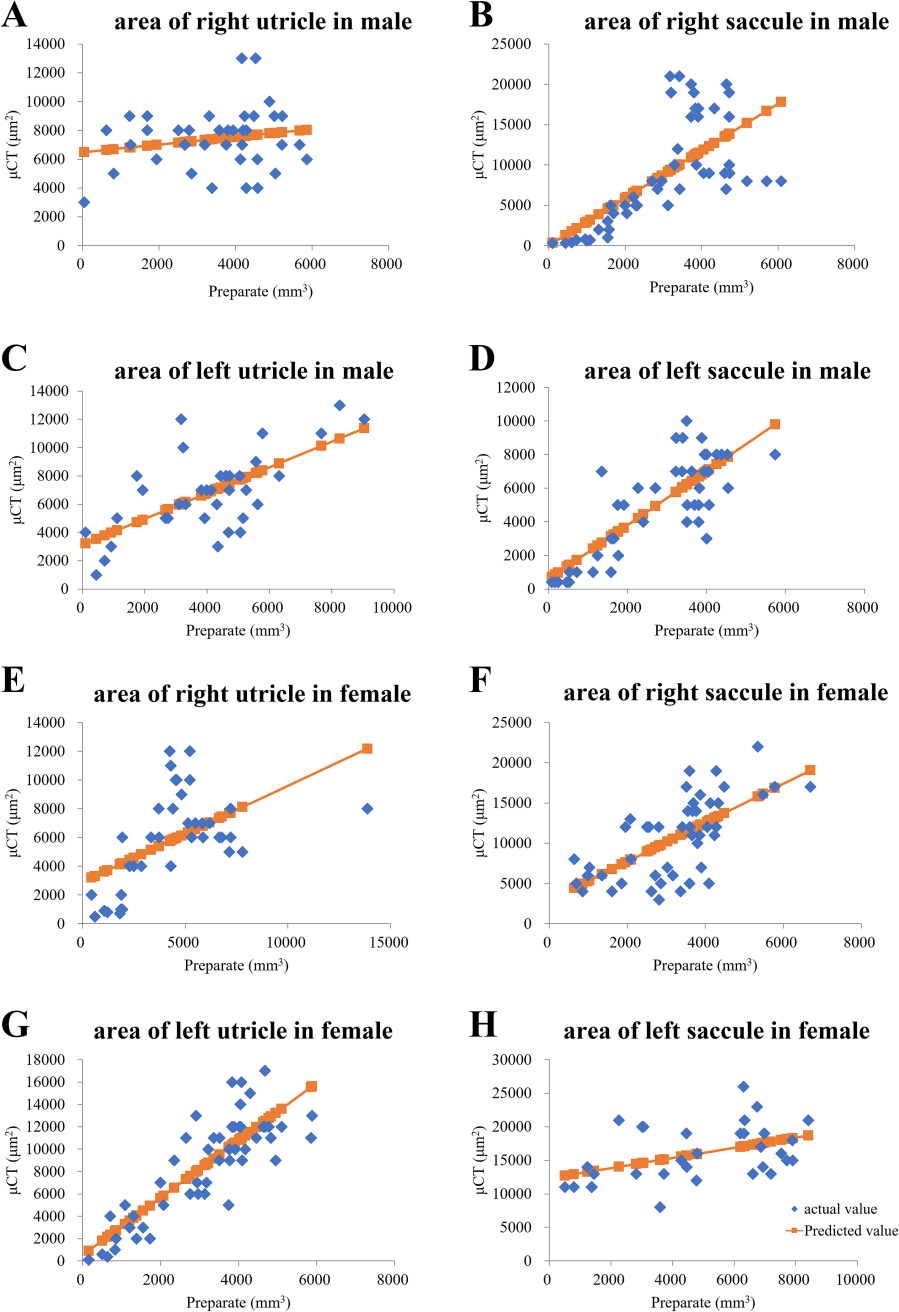
In a young male mouse, the otolith area on HE-stained tissue slides was proportional to that in μCT slices. **(A)** Right utricle area of the male mouse did not exhibit a proportional trend. *p* = 0.26 via regression analysis. **(B)** Right saccule. *p* = 0.00000024 via regression analysis. **(C)** Left saccule. *p* = 0.0000095 via regression analysis. **(D)** Left saccule. *p* = 0.000000000033 via regression analysis. In a young female mouse, otolith area on hematoxylin and eosin (HE)-stained tissue slides was proportional to that in micro-computed tomography (μCT) slices. **(E)** Right utricle. *p* = 0.00074 via regression analysis. **(F)** Right saccule. *p* = 0.00000069 via regression analysis. **(G)** Left utricle. *p* = 0.011 via regression analysis. **(H)** Left saccule. *p* = 0.0000000000000036 via regression analysis.

To confirm the morphology of the utricle and saccule shown in the μCT images, we prepared inner ear sections from the same mice that underwent μCT imaging and compared the μCT and tissue slide images. The μCT images were acquired at a thickness of 10 μm, and the tissue sections were also cut at the same thickness. Tissue images were obtained using a Zeiss Axiocam 208 color microscope (ZEISS AG, Oberkochen, Germany) with a 10 × 20 field of view, and the files were converted into tiff images. The areas of the utricle and saccule in the tissue section images were manually measured using ImageJ software as described previously (Nishihara et al., 2020). The utricle and saccule areas in μCT images were also manually measured using Attractive (PixSpace, Ltd., Fukuoka, Japan). Single regression analysis using the otolith area on tissue images as the explanatory variable and that on μCT images as the response variable revealed correlations in seven of eight samples (Figures 2B–H); however, no correlations were observed in the right utricle of an 8-week-old male mouse (Figures 2A). The otoliths were similarly depicted in both μCT and tissue slide images.

#### 2.2.2 Utricular striola analysis via 3D μCT

As shown on a μCT image (Figure 1D), there was an area in the center of the utricle with a CT value lower than the CT threshold we defined. When we made a 3D model of the utricle, it had a morphology similar to the already known striola. The central part indicated the striola of the utricle. As shown in Figure 1F, the saccule striola could not be identified. Therefore, we attempted to analyze the boundary between the striola and extrastriola of the utricle.

In some cases, no otoconia or small amounts of otoconia were observed at the boundary. An analysis was conducted to quantify this, as described below.

**Figure 3:**
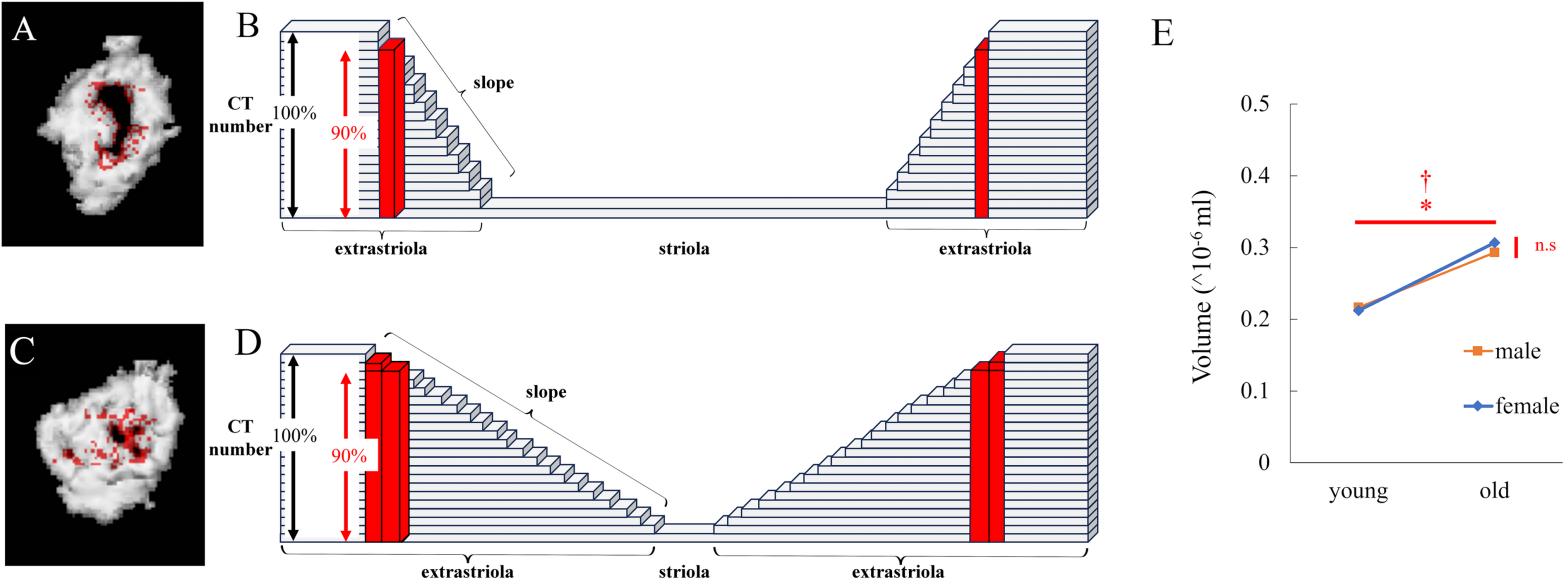
Analysis of the edge of striola via μCT. **(A)** Reconstructed 3D model of the utricle of a young mouse. Red points were detected. The number obtained by multiplying the CT number in the utricle by 0.9 ≤ CT number of the red points < CT number in the utricle. **(B)** Schema for **(A)**. The height of each block indicates the CT number. The height of the black double-headed arrow indicates the threshold CT number to detect the otoliths. As the height of the red block is lower than the threshold, it is recognized as part of the striola and not part of the extrastriola. The height of the red block is higher than that obtained by multiplying the height of the black double-headed arrow by 0.9. Therefore, the red block is part of the edge of the striola, whose CT number is greater than the number obtained by multiplying the height of the black double-headed arrow by 0.9. When the slope indicated by the white triangle is steep, the number of red blocks is low. **(C)** Reconstructed 3D model of the utricle of an old mouse. CT threshold in Figure 1D was multiplied by 0.9, and the 3D model was reconstructed. The volume of the striola increases with a decreasing threshold, as shown in red. **(D)** Schema for **(C)**. The slope indicated by the black triangle is gentle compared to that indicated by the white triangle in **(B)**. Therefore, number of red blocks is higher than that in **(B)**. **(E)** The volume of the edge of the striola is shown by red points. Volume showed a significant difference with age but no significant difference with sex, and no interaction was observed (age, *p* = 0.029; sex, *p* = 0.066; interaction, *p* = 0.34; *n* = 38).

When the threshold was slightly reduced, the volume of the newly detected region (red in Figure 3A) was small when no otoconia were present at the boundary (Figure 3B). However, when a small amount of otoconia was present at the boundary (Figure 3D), the volume of the newly detected part was large (red in Figure 3C).

Next, the volume of the region with CT number < (*α* + *β*)/2 and > (α + β)/2*0.9 (Figures 3A,C) in the utricular striola was determined. The values for α and β were defined in “2.2.1.1 Morphological Analysis of Otoliths via μCT Imaging.” The CT number in the ROI of otolith (utricle and saccule) is α, and the CT number in the ROI of the surrounding tissue is β.

#### 2.2.3 Back-scattered electron-scanning electron microscopy

##### 2.2.3.1 Tissue preparation

**Figure 4:**
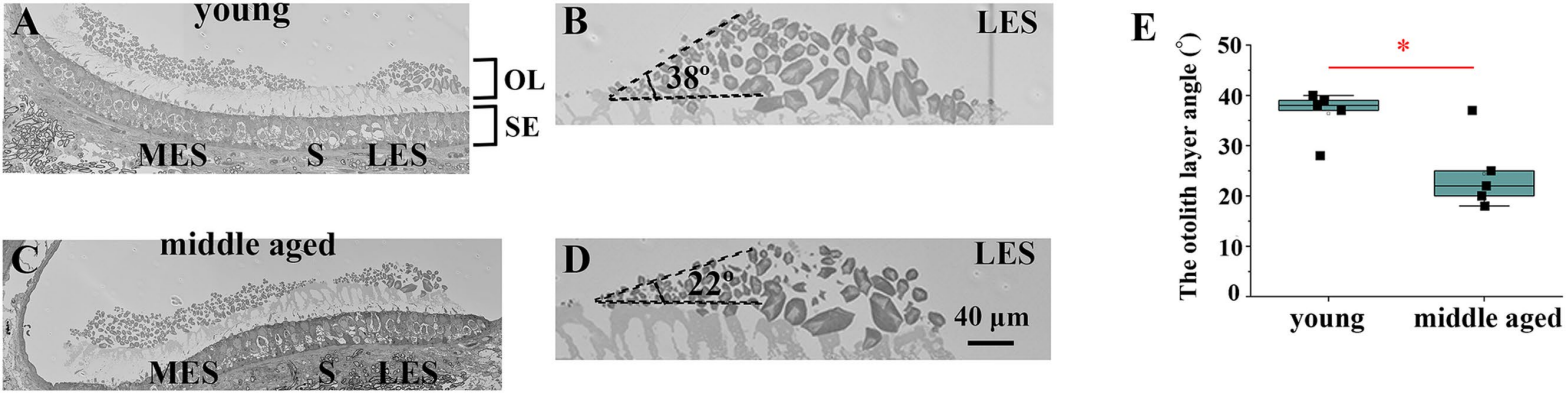
Analysis of the edge of striola via electron microscopy. **(A)** Electron micrograph showing the striola of the utricle in a young mouse. **(B)** Enlarged view of the area around the young mouse’s lateral extrastriola (LES). The angle between LES and striola is preserved to a certain degree. **(C)** Electron micrograph showing the striola of the utricle in a middle-aged mouse. **(D)** Enlarged view of the area around the LES of the middle-aged mouse. The angle between LES and striola is acute. **(E)** Comparison of the otolith layer angle between young and middle-aged mice. Young mice showed significantly larger otolith layer angles than middle-aged mice (*p* = 0.017). OL, otoconial layer; SE, sensory epithelium; MES, medial extrastriola; LES, lateral extrastriola.

Bony labyrinths were acutely dissected from euthanized animals and fixed with a fixative containing 4% paraformaldehyde (EMS, #15710) and 2.5% glutaraldehyde (#17003–05; Nacalai Tesque, Kyoto, Japan) in 0.1 M 4-(2-hydroxyethyl)-1-piperazineethanesulfonic acid. Two hours after fixation at room temperature (25°C), the specimens were washed and immersed in 0.5 M ethylenediamine tetraacetic acid for decalcification and fixed with (1%(w/v) OsO_4_). After fixation, the tissues were dehydrated in a graded series of ethanol solutions (50, 60, 70, 80, 90, 99, and 100%) and embedded in epoxy resin. Ultrathin sections were prepared using an ultramicrotome (Leica UC7, Leica Microsystems Japan, Tokyo, Japan). The sections were collected on cleaned silicon wafer strips for backscattered electron scanning electron microscopy (BSE-SEM) and stained at room temperature with 2% (w/v) aqueous uranyl acetate (20 min) and Reynolds lead citrate (3 min). Images were obtained using an electron microscope (JSM-7900F; JEOL; Figures 4A–D).

### 2.3 Functional changes in otoliths due to aging determined by analyzing the LVOR-induced eye movements

#### 2.3.1 Surgical manipulation

Mice were intraperitoneally injected with a mixture of ketamine (100 mg/kg) and xylazine (10 mg/kg) and anesthetized using a local anesthetic (1% lidocaine). A small incision was made on the skin of the head of each mouse, and a small metal plate with screw holes was fixed at the center of the skull using dental cement (Sun Medical, Shiga, Japan). These surgical manipulations were necessary to measure the eye movements in mice.

#### 2.3.2 Stimulation of linear acceleration in mice

**Figure 5:**
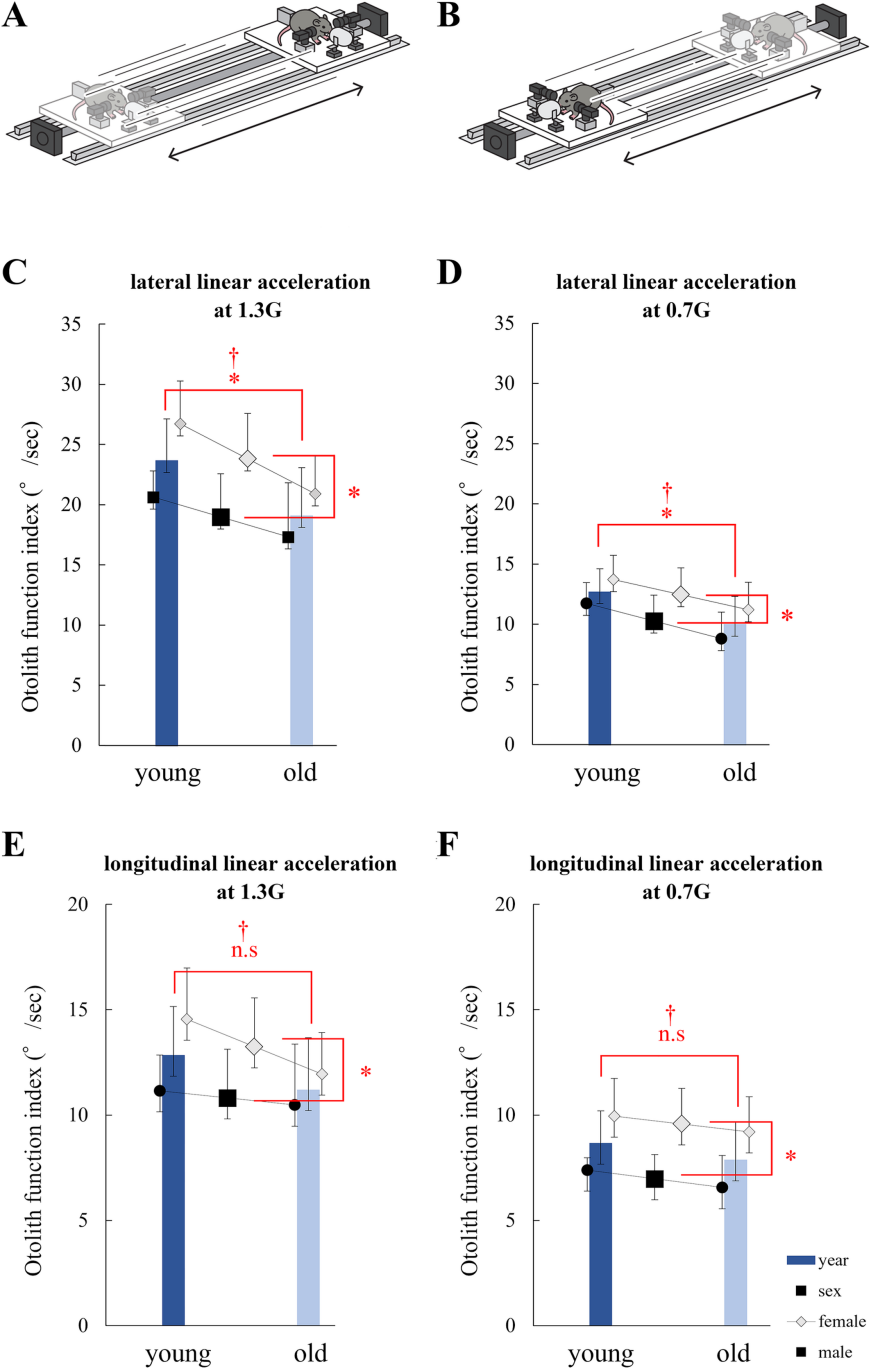
Analysis of eye movements induced by the linear vestibulo–ocular reflex (LVOR). **(A)** Schematic of the linear acceleration applied to mice for utricle stimulation. A mouse was accelerated laterally. **(B)** Schematic of the linear acceleration applied to mice for saccule stimulation. A mouse was accelerated longitudinally. **(C)** Otolith function index during 1.3G lateral stimulation. Significant differences in age and sex were observed among the groups. No interactions were observed. (age, *p* = 0.014; sex, *p* = 0.010; interaction, *p* = 0.48; *n* = 40). **(D)** Otolith function index during 0.7G lateral stimulation. Significant differences were observed in age and sex among the groups. No interactions were observed (age, *p* = 0.015; sex, *p* = 0.045; interaction, *p* = 0.84; *n* = 40). **(E)** Otolith function index during 1.3G longitudinal stimulation. Significant differences were observed in age, but not in sex. No interactions were observed (age, *p* = 0.17; sex, *p* = 0.045; interaction, *p* = 0.42; *n* = 40). **(F)** Otolith function index during 0.7G longitudinal stimulation. Significant differences were observed in age and sex among the groups. No interactions were observed (age, *p* = 0.30; sex, *p* = 0.001; interaction, *p* = 0.95; *n* = 40).

First, the mouse was placed on a linear sled with a plastic cylindrical container and computer-controlled motor (Figures 5A,B). As described above, a metal plate was attached to the mouse’s head to restrain its movements. The metal plate and sled were fixed such that the sled and mouse head did not separate while the sled moved. The sled was fixed to a straight stainless-steel rail parallel to the ground and moved in a reciprocating linear motion. Sleds and rails were manufactured by BioMedica Corporation (Osaka, Japan). The sled was accelerated and decelerated from the right end, traveled 1800 mm to the left and back ends, and reciprocated five times. The sled was stationary at both the right and left ends for approximately 0.3 s, and the maximum acceleration and speed were set at the following two levels: 1.3 G (3.25 m/s) and 0.7 G (3.06 m/s). The sled could freely change its angle to the rail, and the experiment was performed using a mouse placed horizontally and vertically relative to the rail. In the transverse orientation, the mice moved right and left, and linear acceleration was applied in the interaural direction. In the longitudinal orientation, the mice were moved in the front-back direction, and linear acceleration was applied in the cephalonasal direction. The order of the horizontal and vertical orientations and the acceleration settings were randomized. The experiments were conducted in the dark.

#### 2.3.3 Analysis of LVOR-induced eye movements

A high-speed infrared camera (sampling rate 240 Hz; STC-CL338A; Centec Corporation, Kanagawa, Japan) was used to record eye movements during the exercise. Images of both eyes were acquired using StreamPix software (NorPix, Montreal, Canada). The camera was placed directly next to the eyes to analyze eye movements during movement. Since pupil dilation in the dark makes analysis difficult, eye drops (1% pilocarpine hydrochloride, Nippon Tenyaku Kenkyusho, Nagoya, Japan) were used to constrict the pupils during the experiment. Markers were set on a sled to record the movements of the mice during translational linear acceleration stimulation, and the movements of the markers were recorded using a high-speed infrared camera (STC-CL338A). The acquisition of marker images was synchronized with eye images using StreamPix software (Stream-Pix). The center coordinates of the markers were extracted by binarizing the marker images. The position of each mouse was then calculated using these coordinates.

The 240-Hz movies showing eye movements induced by LVOR were analyzed using an algorithm developed in our laboratory (Imai et al., 2016). Details of the method of three-dimensional analysis of eye movements are provided in Supplementary Figure 1. The position of the eyeball was expressed as a vector around its axis, the length of which was proportional to the rotation angle. The reference position was the eyeball at rest. The X, Y, and Z component were mainly reflected by the torsional, vertical, and horizontal components, respectively.

As reported by Harada et al., the amplitude of the right eye movement is similar to that of the left eye movement. Therefore, it is sufficient to analyze only one eye movement when analyzing the amplitude of eye movements. The evaluation indices used in this experiment were as follows:

Otolith function index = ([maximum positive shift angle of the vertical component of the left eye]–[maximum negative shift angle of the vertical component of the left eye])/2.

**Figure 6:**
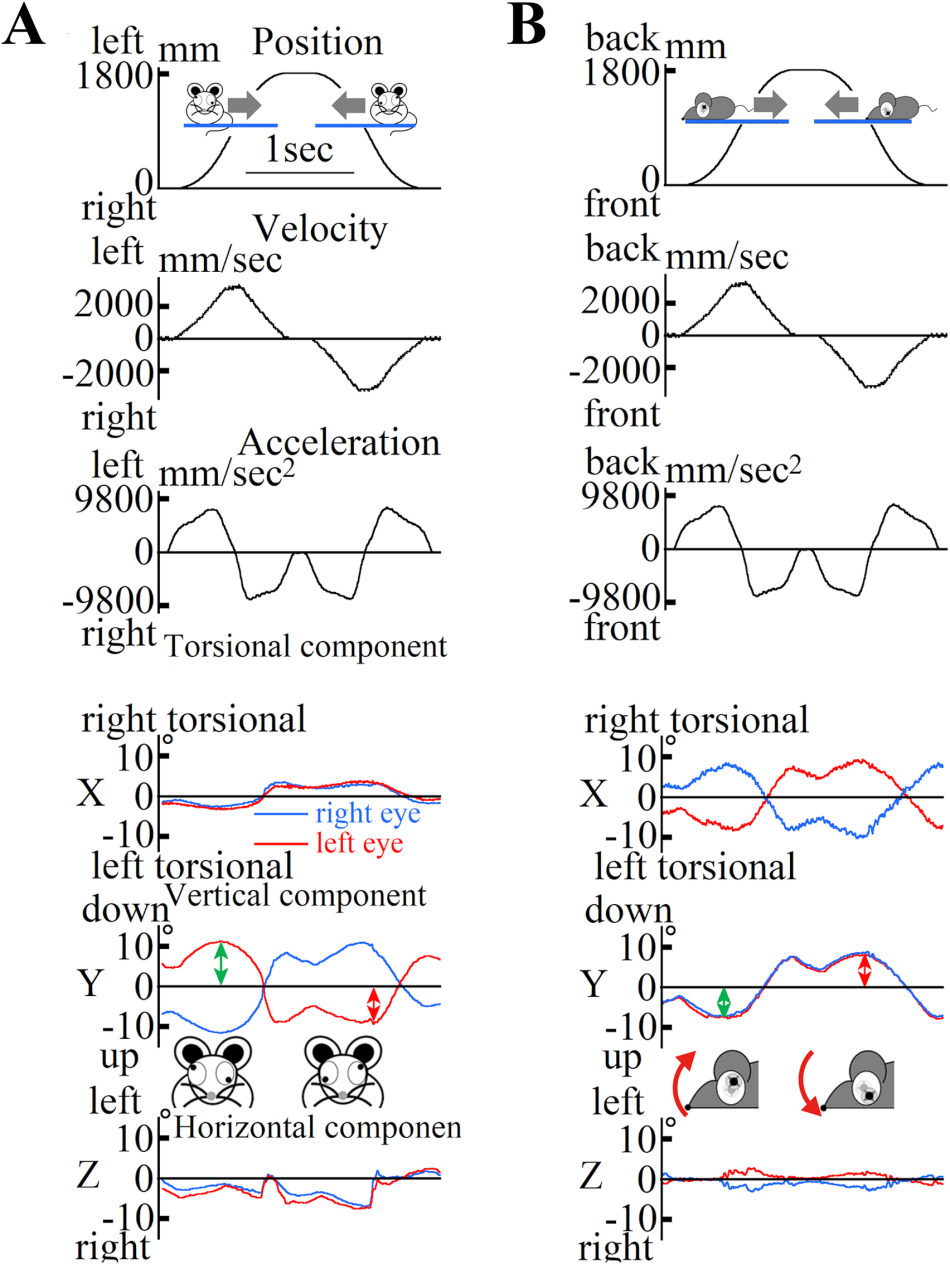
**(A)** Changes in mouse position during lateral translational motion. First column: Mouse position data; Second column: Mouse velocity data; Third column: Mouse acceleration data in the interaural direction. The mouse was rotated left and right over a one-way length of 1800 mm over five round trips. The mouse remained at the leftmost and rightmost edges for 0.3 s. Data were collected during the third and fourth trips. The mouse moved at a maximum acceleration of 1.3 G. Three-dimensional data from both eyes of the mouse showed inappropriate disconjugate vertical components during lateral translational motion in the dark. We recorded the movements of both the eyes of the mouse using high-speed cameras and analyzed the recorded images using our offline computer image analysis system (Imai et al., 2016). The data represent the three-dimensional movement of both eyes during motion, as shown in **(A)**. In this study, eye movements were described three-dimensionally by the axis angle, which characterized the eye positions around a single rotation. The three-dimensional coordinates of the eye were as follows: the X-axis parallel to the interaural axis (positive left in the left eye; positive right in the right eye), the Y-axis parallel to the naso-occipital axis (positive backward in the left eye; positive forward in the right eye), and the Z-axis normal to the X–Y plane (positive upward; see insert). The X, Y, and Z components reflect the torsional, vertical, and horizontal components, respectively. The direction of rotation was described from the perspective of the mice. For the X-component, “right torsional” and “left torsional” indicate that the superior pole of the eyeball rotated to the right and left, respectively. The main component was the vertical component, and the waveform (second column) was similar to that of the mouse acceleration [third column in **(A)**] but not to that of the mouse position [first column in **(A)**]. Vertical eye movements were disconjugated (during rightward acceleration, the left eye moved upward, and the right eye moved downward). Conjugated horizontal eye movements compensating for the lateral translational motion observed in humans were not observed in mice. All the mice exhibited disconjugated vertical eye movements under dark conditions. **(B)** Changes in mouse position during back-and-forth translational motion. First column: mouse position data, second column: mouse velocity data, third column: mouse acceleration data in the naso-occipital direction. The mouse was moved back and forth over a one-way length of 1800 mm for five round trips. The mice remained at the leftmost and rightmost edges for 0.3 s. Data were recorded during the third and fourth trips. The mice moved with a maximum acceleration of 1.3 G. The waveforms of the position, velocity, and acceleration are similar to those shown in **(A)**. Three-dimensional data from both eyes of the mouse during back-and-forth translational motion under dark conditions. These data are three-dimensional position data for the movement of both eyes during the motion shown in **(A)**. Eye movements were described three-dimensionally in the same manner as described in **(A)**. Disconjugated torsional, vertical, and horizontal eye movements were also observed. These waveforms had a shape similar to that of the mouse acceleration [third column in **(A)**], but not to that of the mouse position [first column in **(A)**]. This indicates that eye movement responds to linear acceleration in the naso-occipital direction and does not compensate for the motion of the mouse to stabilize its gaze in space.

The above equation was analyzed for the period of eye movement during one repetitive eye movement cycle to obtain the otolith function index, which was statistically compared by averaging the three highest values of the five repetitive eye movement cycles to obtain the actual otolith function index. Representative data of the position, velocity, acceleration, and eye movements during acceleration in old mice are shown in Figure 6.

### 2.4 Statistical analyses

Shapiro–Wilk and Kolmogorov–Smirnov tests were conducted to evaluate the normality of all variables. When normality was detected, a two-way analysis of variance was used. When an interaction effect was observed, the Bonferroni test was used to evaluate the differences between the groups.

Statistical analyses were conducted using SPSS v.25.0 (IBM Corp., Armonk, NY, United States) and GraphPad Prism v.8.00 for Windows (GraphPad Software, La Jolla, California, United States). Statistical significance was set at *p* < 0.05.

## 3 Results

### 3.1 Morphological changes in otoliths due to aging

#### 3.1.1 Changes in volume determined via 3D μCT

Little correlation was observed between the utricle volume, age (*p* = 0.11), and sex (*p* = 0.72). No significant interaction was observed (*p* = 0.76). Little correlation was observed between the saccule volume, age (*p* = 0.52), and sex (*p* = 0.44). No significant interactions were detected (*p* = 0.87; Figures 1H,I). The results indicated no statistically significant differences in otolith volumes between ages and sexes.

#### 3.1.2 Changes in values determined via 3D μCT

Utricle CT number was correlated with age (*p* = 0.001), but not with sex (*p* = 0.32). Little interaction was observed (*p* = 0.58). Saccule CT number was correlated with age (*p* = 0.026) and sex (*p* = 0.0030). Significant interactions were detected (*p* = 0.034; Figures 1J,K).

To account for interactions in saccule CT numbers, as shown in Figure 1K, we performed the Bonferroni-corrected test using four groups: young male, young female, old male, and old female mouse groups. *p* values for comparisons between the two groups are described below.

Between young male and young female mice: *p* = 0.99; between old male and old female mice: *p* = 0.0016; between young male and old male mice: *p* = 0.0074. And between young female and old female mice: *p* = 0.99. We observed significant differences between old male and old female mice and between young male and old male mice.

As discussed below, the CT number correlated with density. Thus, the results in this section refer to the density of the otoliths. The results indicated that the density of otoliths significantly decreases with age. The density of otoliths in male mice tended to be smaller than that of otoliths in female mice. At least in saccules, otolith density in male mice was significantly smaller than in female mice (Figures 1J,K).

#### 3.1.3 Utricular striola analysis via 3D μCT

For the volume of the newly detected part at the boundary between the striola and extrastriola of the utricle, as shown in Figure 3E, significant differences were observed in age (*p* = 0.029), but not in sex (*p* = 0.066). The interactions were not significant (*p* = 0.34).

The results indicated that the volume of the part at the boundary between striola and extrastriola was significantly larger in old mice than in young mice. As discussed below, this results suggested that the part of boundary had become blurred with aging. Considering the electron microscopy results, this suggests that the morphology of the striola may be collapsed, which will be discussed in more detail in the discussion.

### 3.2 Electron microscopy

Representative images of the utricles were obtained using an electron microscope (Supplementary Figure 2). As shown in Figures 4A,B, the striola of young mice had a thinner otoconial layer than the extrastriola. This is consistent with previous reports that the otolith layer is thinner on the striola (Lim, 1971; Lim, 1979; Lindeman, 1969). However, in the striola of middle-aged mice, the otolith layer angle, which is the angle between the lateral extrastriola (LES) and the basal plane of the striola, was reduced (Figures 4C,D). We measured the otolith layer angles in all mice (Figure 4E). Significant differences in the otolith layer angles were observed between the young and middle-aged mice (*p* = 0.017).

Considering the result of utricular striola analysis via 3D μCT, the change in the LES-striola angle may indicate the collapse of the structure of boundary between the LES and the striola. These results were consistent with what would be expetcted from the utricular striola analysis via 3D μCT.

### 3.3 Otolith function index

During lateral linear acceleration (Figure 5A), disconjugate vertical eye movement was observed in the Y component (Figure 6A). The value of the otolith functional index in the analyzed mouse during lateral linear acceleration was the sum of the amplitude indicated by the green double-headed arrow and that indicated by the red double-headed arrow in Figure 6A. During longitudinal linear acceleration (Figure 5B), conjugate vertical eye movements were observed in the Y component (Figure 6B). The value of the otolith function index of this mouse during longitudinal linear acceleration was the sum of the amplitude indicated by the green double-headed arrow and that indicated by the red double-headed arrow in Figure 6B. We measured the otolith function indices in all mice.

During lateral linear acceleration at 1.3G, significant differences in otolith function index were observed between the young and old mice (*p* = 0.014) and between the male and female mice (*p* = 0.010). No significant interactions were observed (*p* = 0.48; Figure 5C). At 0.7G, significant differences in otolith function index were observed between the young and old mice (*p* = 0.015) and between the male and female mice (*p* = 0.045). No significant interactions were observed (*p* = 0.84; Figure 5D).

During longitudinal acceleration at 1.3G, no significant differences in otolith function index were observed between the young and old mice (*p* = 0.17) and between the male and female mice (p = 0.045). No significant interactions were observed (*p* = 0.83; Figure 5E). During longitudinal acceleration at 0.7G, no significant differences in otolith function index were observed between the young and old mice (*p* = 0.30), but significant differences were observed between the male and female mice (*p* = 0.0010). No significant interactions were observed (*p* = 0.95; Figure 5F).

The otolith function index of the lateral acceleration stimulus reflects utricle function, and the otolith function index of longitudinal acceleration reflects the function of the saccule. The results indicate that otolith function tends to decrease with age. In particular, utricle function decreased significantly with age and tended to be smaller in males than in females. These results were not entirely consistent with the μCT and electron microscopy results, but showed similar tendency to the μCT and electron microscopy results.

## 4 Discussion

In this study, we demonstrated that the utricle functions in mice declined with age by analyzing their eye movements during linear translation (Figure 5). We also showed age-related morphological changes in the otolith organ, including the decrease in the density of otoconia on the otolithic membrane (Figures 1J,K) and the collapse of the structure of the otoconial layer with age. The collapse of the structure of the utricle striola could be observed using CT and electron microscopy images (Figures 3, 4). These results suggest that age-related morphological changes are one of the causes of the decline in otolith function.

A decrease in the density of otoconia may have caused a decrease in the function of the otolith organs. When linear acceleration was applied during the translational movement of mice, the otoconia was subjected to an inertial force whose direction was opposite to that of the linear acceleration. The kinocilia and stereocilia of hair cells under the otoconia were tilted by the inertial force of the otoconia, which induced the depolarization and hyperpolarization of hair cells (Eatock et al., 1987; Eatock and Lysakowski, 2006). When the density of otoconia decreased, a decrease in otoconia could potentially affect the speed or extent at which the cilia are moved, hence affecting the hair cell response and reducing the occurrence of depolarization and hyperpolarization. These changes may partially explain the decline in otolith function; however, other likely changes (e.g., changes in hair cells, afferents, efferents and synaptic properties) that could underlie alterations observed with aging must also be considered. These are discussed in more detail below.

Collapse of the structure of the otoconial layer or collapse of the structure of the striola induced a decline in the function of the otolith organ. Hair cells are oriented with their kinocilia positioned toward each other in the utricle, but they are positioned away in the saccule, and the cell boundary separating these groups is called the line of polarity reversal (Hama, 1969; Deans, 2013; Nam et al., 2019; Ono et al., 2020; Jia et al., 2023). Linear head motion excites all hair cells on one side and inhibits the hair cells on the opposite sides of the line of polarity reversal. When the structure of the striola was collapsed, the sensory information input about acceleration may not work well and may lead to a decrease in otolithic organ function.

In this study, we assessed the function of otoliths by analyzing eye movements during linear translational movements. Information on the linear translational movement of the mice was detected using otoliths. Information is transmitted to primary neurons and then to the oculomotor nucleus, trochlear nucleus, and abducens nucleus, resulting in an eye movement called the VOR. Our previous study confirmed that mice stimulated by linear acceleration exhibited the VOR (Harada et al., 2021). This VOR is called the LVOR. The utricle-evoked LVOR can be observed during lateral linear translational movement (Figures 5C,D), and the saccule-evoked LVOR can be observed during longitudinal linear translational movement (Figures 5E,F). Later linear translational movement (Figures 5C,D) mainly stimulates the utricle, while the longitudinal movement mainly stimulates the saccule. Vertical eye movement can be seen in the utricle-evoked LVOR and saccule-evoked LVOR (Harada et al., 2021). Therefore, the function of the utricle can be assessed by analyzing the amplitude of the vertical eye movement during lateral linear acceleration, and the function of the saccule can be assessed by analyzing the amplitude of the vertical eye movement during longitudinal translational movement. This study refers to the amplitude as the otolith function index. When the otolith function index is low, the otoliths (utricle and saccule) are impaired. As shown in Figure 5, the value of the utricle function index in old mice was smaller than that in young mice, indicating that utricle function is impaired in old mice compared to young mice, and utricle function declines with aging; however, the Otolith Function Index may be affected by functioning of otoliths as well as other factors (e.g., muscles as well as central neurons and synapses), particularly with aging. This point is discussed in more detail below.

Some studies have used μCT to visualize and measure the volume of the otoliths (Honda et al., 2015; Nishihara et al., 2020). We compared the μCT images with the tissue images and confirmed that the otoliths could be delineated (Figure 2). The CT number reflects the X-ray attenuation coefficient of an image voxel. As otoliths have a high attenuation rate of X-rays, they appear as high-attenuation areas on the CT image, and the CT number of the area is high (Koezuka et al., 2022; Bai et al., 2023). The denser the otolith, the greater the CT number. Therefore, when the density of the otoliths decreases, the CT number also decreases. As shown in Figure 1, the CT number in old mice was lower than that in young mice, indicating that the density of otoliths was lower in old mice than in young mice and that the density of otoliths declined with age.

Electron microscopy images showed that the otolith layer angle decreased with age in the mouse utricles (Figure 4). The otolith layer angle is the angle between the LES and the basal plane of the striola. The angle ranges from 0° to 90°. When the angle was 0°, no boundary was observed between the striola and LES. However, striola and LES regions are clearly distinguishable when the angle is 90°. Therefore, a slight angle indicated the collapse of the striola morphology. As shown in Figure 4, the angles in old mice were significantly smaller than those in young mice. Electron microscopy revealed that the striola morphology of the old mice collapsed more than that of the young mice.

The CT number of the extrastriola region was significantly higher than that of the striola. As the CT number decreases from the extrastriola to the striola boundary, as shown in the schema in Figure 3B, the striola and striola region can be easily distinguished by the threshold CT number. Even when the threshold number was slightly reduced, the areas judged as striola and striola region remained almost unchanged. Therefore, when the threshold number was reduced to 90%, the areas judged as striola increased; however, the increase was slight (Figure 3B). When the morphology of the striola collapsed and the boundary between the striola and extrastriola became unclear, the increase was significant when the threshold number was reduced to 90% (Figure 3D). As shown in Figure 3E, the increase in old mice was significantly greater than that in young mice. CT image results indicate that the morphology of the striola collapsed more in old mice than in young mice, which is consistent with the electron microscopy results (Figures 3E, 4E).

Considering the results of utricular striola analysis via 3D μCT, the change in the LES-striola angle may imply a collapse of the structure of boundary between the LES and the striola. A previous study reported that otoconia, which are CaCO_3_ biominerals precipitated around a proteinaceous core, are embedded in the gelatinous membrane and maintained in place by strands of noncollagenous extracellular matrix proteins that resemble beads on a string (Hughes et al., 2006). Therefore, we hypothesize that the structure of the gelatinous membrane and noncollagenous extracellular matrix proteins changes with age, resulting in an increase in floating otoconia debris, a decrease in otoconia on the LES, and a decrease in the LES-striola angle; however, we are not aware of any study describing age-related changes of gelatin membranes or noncollagenous extracellular matrix proteins in the otoliths, which should be examined in future research.

Overall, this study showed that aging causes utrcile dysfunction and decreasing the density of otoliths and collapsing the utricle striola morphology, and showed possible correlations.

### 4.1 Differences between male and female mice

The otolith function index of LVOR was significantly smaller in males than in females for both the utricle and saccule (Figures 5C–F). In previous studies, C56BL/6 N female mice performed better than male mice in the rotarod test (McFadyen et al., 2003; Bothe et al., 2004; Ashworth et al., 2015), indicating that female mice have better otolith function than male mice. Our μCT experiment showed that females had significantly higher CT numbers in saccule than the male mice, but no significant differences were observed in the CT numbers in utricle. Female mice exhibited higher CT numbers and density of the otolith than the male mice (Figure 1). These results suggest that female mice exhibit higher otolith density may partially explain better otolith function than male mice.

A previous study showed that there was no difference in VsEP response between males and females in CBA/Caj mice (Mock et al., 2011). Differences among mouse types may be a factor, but it is difficult to determine the cause of the sex difference.

### 4.2 Limitations

One of the potential biases of this study is related to the fact that the groups of young and old mice were not maintained in the same place and were not exposed to the same experimental conditions. For example, it is known that fish in rearing tanks influenced by various physical and social conditions are more likely to form larger, more deformed, and lower-density otoliths than normal otoliths, called vaterites (Delaval et al., 2021). Mice born to manganese-deficient parents exhibit reduced or no otoconia (Erway et al., 1970). In this study, we used mice without walking balance problems. The manganese concentration in the diet was 0.0097% for the young mice and 0.0105% for the middle-aged and old mice, with little difference between them. It is unclear whether similar phenomena occur in mice as in fish; however, the fact that groups of young and old mice were fed in different environments should be noted.

In this study, electron microscopy experiments were performed on young and middle-aged mice, but not on older mice because of the following reasons: First, we performed electron microscopy, μCT, and eye movement analysis during linear acceleration experiments on young and middle-aged mice and found significant differences in the electron microscopy results (Figure 4) but no significant differences in μCT and eye movement analysis during linear acceleration experiments (Supplementary Figures 3, 4); however, as a trend of age-related changes was expected, we performed μCT experiments and after analyzing the LVOR-induced eye movements, we found significant differences. Thus, because electron microscopy confirmed significant differences between young and middle-aged mice, we did not perform additional experiments in old mice.

When preparing slices for electron microscopy, we carefully performed the same procedure on each mouse for preventing the individual differences; however, slices cannot be perfectly reproduced at the exact same angle or location between individuals, which may have introduced bias.

Longitudinal acceleration causes not only vertical eye deviation but also torsional eye deviation, which was confirmed in the eye movement analysis in the present experimental results (Figure 6B). We analyzed the vertical, horizontal, and torsional eye deviations that occurred during longitudinal acceleration. The results showed that the torsional and vertical components were larger than the horizontal component, and the amplitudes of the torsional and vertical components were almost the same; therefore, either the torsional or vertical components were the optimal parameters under these conditions. When analyzing eye movements from video recordings, the vertical component could be analyzed simply by measuring the two-dimensional coordinates of the “pupil center” in the video; however, to analyze the torsional component, the two-dimensional coordinates of both the center of the pupil in the video and the “iris freckle” were measured. In the present study, the eyes of some mice showed white staining, possibly because of aging, and it was sometimes difficult to distinguish freckles in the iris. Thus, the results torsional and vertical component analyses were equivalent, but the torsional component required more manufacturing processes for analysis than the vertical component, so the analysis of the vertical component was adopted.

In this study, we varied the number of CT thresholds to distinguish between the otoliths and other areas of mice. This is because the number of CT thresholds in young mice is much higher than that in old mice. If the same threshold number is used when analyzing old mice as that used for young mice, it will be impossible to distinguish between the otolith and other areas in μCT.

Otolith function is affected by various factors, such as otoconia morphology and quantity, hair cells involved in otoconia movement, primary nerves transmitting information from the hair cells to the center, and vestibular nucleus receiving information from the primary nerve and other organs related to output (such as VOR) that process information from the otolith organ.

Several studies reported otolith function decreasing and otolith morphology changing with age, either directly or indirectly (Ross et al., 1976; Nakayama et al., 1994; Lyon and Davis, 2002; Iwasaki et al., 2009; Agrawal et al., 2012; Maheu et al., 2015; Wan et al., 2019). Zheng et al. revealed that C57BL/6 J mice displayed loss of the hair cells and spiral ganglion neurons and increased hearing thresholds by 12 months of age (Zheng et al., 1999). A different study claimed that aged C57BL/6 mice display an age-related decline in the density of spiral ligament and stria vascularis (Ichimiya et al., 2000). Neuroanatomical studies of the peripheral vestibular end-organs in older people have consistently shown attrition of neural and sensory cells as a function of age (Bergström, 1973; Rosenhall, 1973; Richter, 1980). One study opined that “although there is evidence for age-related hair cell loss and neuronal loss in Scarpa’s ganglion and the vestibular nucleus complex (VNC), it is not entirely consistent. It is concluded that, at present, it is difficult, if not impossible, to relate the neurochemical changes observed to the function of specific VNC neurons and whether the observed changes are the cause of a functional deficit in the VNC or an effect of it” (Smith, 2016). A different study showed the age-related changes included variation in fiber size, increased endomysial fibrous tissue and increased endomysial adipose tissue, and loss of myofibrils in humans (McKelvie et al., 1999). Several other external factors affecting VOR have been reported. A long-lasting visuo-vestibular mismatch leads to changes in synaptic transmission and intrinsic properties of central vestibular neurons in the direct VOR pathway in mice (Carcaud et al., 2017). Selective silencing of afferents by electrical stimulation alters VOR responses (Minor and Goldberg, 1991); furthermore, extraocular proprioception is important for fixation of the eye position in spacial relation to the head, and loss of the sensation readily induces anticompensatory oculomotor response to head movement (Kashii et al., 1989). While it is unlikely that all of the above are closely related to the results of the presnet study, it should be noted that otolith function is not solely influenced by changes in otolith morphology but is determined by multiple factors.

Here, we showed that the morphology and density of otoconia decreased with age, and the function of otoliths decreased with age. These factors may be correlated. Age-related effects on hair cells, primary nerves, and vestibular nerve nuclei are associated with otolithic organ dysfunction. Paplou et al. reported that the density of hair cells around the striola of the utricle decreases with age in B6 mice (Paplou et al., 2023). To improve age-related otolith dysfunction, age-related changes should be reversed in the hair cells, primary nerves, and vestibular nerves. However, reversing age-related changes requires regeneration technology, which is too complex and time-consuming for practical use. However, reduction in otoconia volume can be resolved by further understanding the process of otoconia formation and its influence; this is a much more viable approach than treatment using regeneration technology. Overall, the results of this study provide valuable information on age-related otolithic organ dysfunction. In conclusion, this study demonstrated that utricle function and otolith density decreased with age in mice.

## Data Availability

The raw data supporting the conclusions of this article will be made available by the authors, without undue reservation.
