# Supplementary material for: Effects of aging on otolith morphology and functions in mice
Source: Front Neurosci. 2024 Oct 16;18:1466514. doi: 10.3389/fnins.2024.1466514 (PMC11521974; doi:10.3389/fnins.2024.1466514)
Supplement: Supplementary file 1 [file Data_Sheet_1.DOCX]

Supplementary Material

# Three-Dimensional Analysis of Eye Movements

In this study, eye movements were three-dimensionally analyzed using rotation vectors characterizing the eye position around a single rotation (Supplementary Figure 1A). Any eye position can be characterized by rotating the eye from the reference position along a single axis. Head coordinates to analyze the left eye were reconstructed in three dimensions as follows: the X-axis was parallel to the interaural axis (positive left), the Y-axis was parallel to the naso-occipital axis (positive backward), and the Z-axis was normal to the X–Y plane (positive upward; Supplementary Figure B). Head coordinates to analyze the right eye were reconstructed in three dimensions as follows: the X-axis was parallel to the interaural axis (positive right), the Y-axis was parallel to the naso-occipital axis (positive forward), and the Z-axis was expected to the X–Y plane (positive upward). The eye movement movies were converted to 644 × 484-pixel JPEG images and analyzed using an algorithm developed by our laboratory (Imai T et al. PLoS One (2016) 11:e0152307). The two-dimensional coordinates of the center of the pupil (yp zp) and iris freckle (yi zi) in the images were determined (Supplementary Figure C) (Imai T et al. PLoS One (2016) 11:e0152307). The edge of the pupil was also detected and approximated using an ellipse. Then, the minor and major axes of the ellipse were determined. The center of eye rotation (*o*) (yc zc) on the image plane was determined as the intersection of the extensions of the minor axes (Supplementary Figure D) (Imai T et al. PLoS One (2016) 11:e0152307). After determining the center of eye rotation, we calculated the radius of rotation at the center of the pupil (*R*) using the following formula:

$$R\sqrt{1-\left( {the length of minor axis}/{the length of major axis} \right)^{2}}=d$$

Here, *d* is the length between *o* and the center of the pupil ellipse, *p* (Supplementary Figure E). Next, we calculated the length of the radius of rotation of an iris freckle (*R’*). We reconstructed the three-dimensional coordinates at the center of the pupil and iris freckle in the head-fixed coordinate system as $\left( \begin{matrix} \sqrt{R^{2}-\left( yp-yc \right)^{2}-\left( zp-zc \right)^{2}} & yp-yc & zp-zc \end{matrix} \right)$ and $\left( \begin{matrix} \sqrt{{R'}^{2}-\left( yi-yc \right)^{2}-\left( zi-zc \right)^{2}} & yi-yc & zi-zc \end{matrix} \right)$.

Relationships between the three-dimensional coordinates at the center of the pupil and iris freckle, compared between the test and reference positions, were used to calculate the rotation vector of the eye position **r** (Haslwanter T. Vision Res (1995) 35:1727-39). The reference position was defined as the eye position when the head of the mouse remained upright. X, Y, and Z components of the eye position axis angle primarily reflected the roll, pitch, and yaw components, respectively (Supplementary Figure B). The direction of rotation is described from the perspective of the mouse. For the X-component, “right torsional” and “left torsional” indicated that the superior pole of the eyeball rotated toward the right and left ears, respectively. Moreover, the Euler angle parameter, indicated as 2 × tan^-1^ (magnitude of rotation vector, **r**), was used to represent the eye position and velocity as an axis–angle representation (Schnabolk C et al. J Neurophysiol (1994) 71:623-638).

The accuracy of this method for analyzing eye rotation vectors has been previously demonstrated (Imai T et al., 2016, PLoS One 11:e0152307) . The accuracy of our video-oculography method was confirmed by matching the analyzed angle of the eyeball simulator with a previously set value (Imai T et al. Acta Otolaryngol (1999) 119:24-28) and by matching the analyzed angle with that determined using a scleral search coil system in humans (Imai T et al. Auris Nasus Larynx (2005) 32:3-9). Additionally, we confirmed the absence of torsional cross-talk between the horizontal and vertical components of the analyzed data when the rotated angle was between −40° and 40° (Imai T et al. Acta Otolaryngol (1999) 119:24-28).

# Preliminary μCT and Linear Vestibulo–Ocular Reflex (LVOR) Experiments

For the preliminary μCT and LVOR experiments, 56-week-old female mice were used. Hereafter, 8-week-old mice will be referred to as young mice and 56-week-old mice as middle-aged mice.

## Three-dimensional (3D) μCT

CT was performed on eight ears of four 56-week-old female mice. After confirming normality in all groups using the Shapiro–Wilk test, the Mann–Whitney *U* test was performed using young and middle-aged mice. The factors analyzed were utricle volume, utricle CT number, saccule volume, and saccule CT number. No significant differences were observed in any of these factors between the two groups.

## LVOR

The LVOR experiments were performed on four 56-week-old female mice. After confirming normality in all groups using the Kolmogorov–Smirnov test, the Mann–Whitney *U* test was performed on young and middle-aged mice. The factors analyzed were lateral acceleration at 1.3G, lateral acceleration at 0.9G, longitudinal acceleration at 1.3G, and longitudinal acceleration at 0.9G. No significant differences were observed in any of these factors between the two groups.

# Supplementary Figures


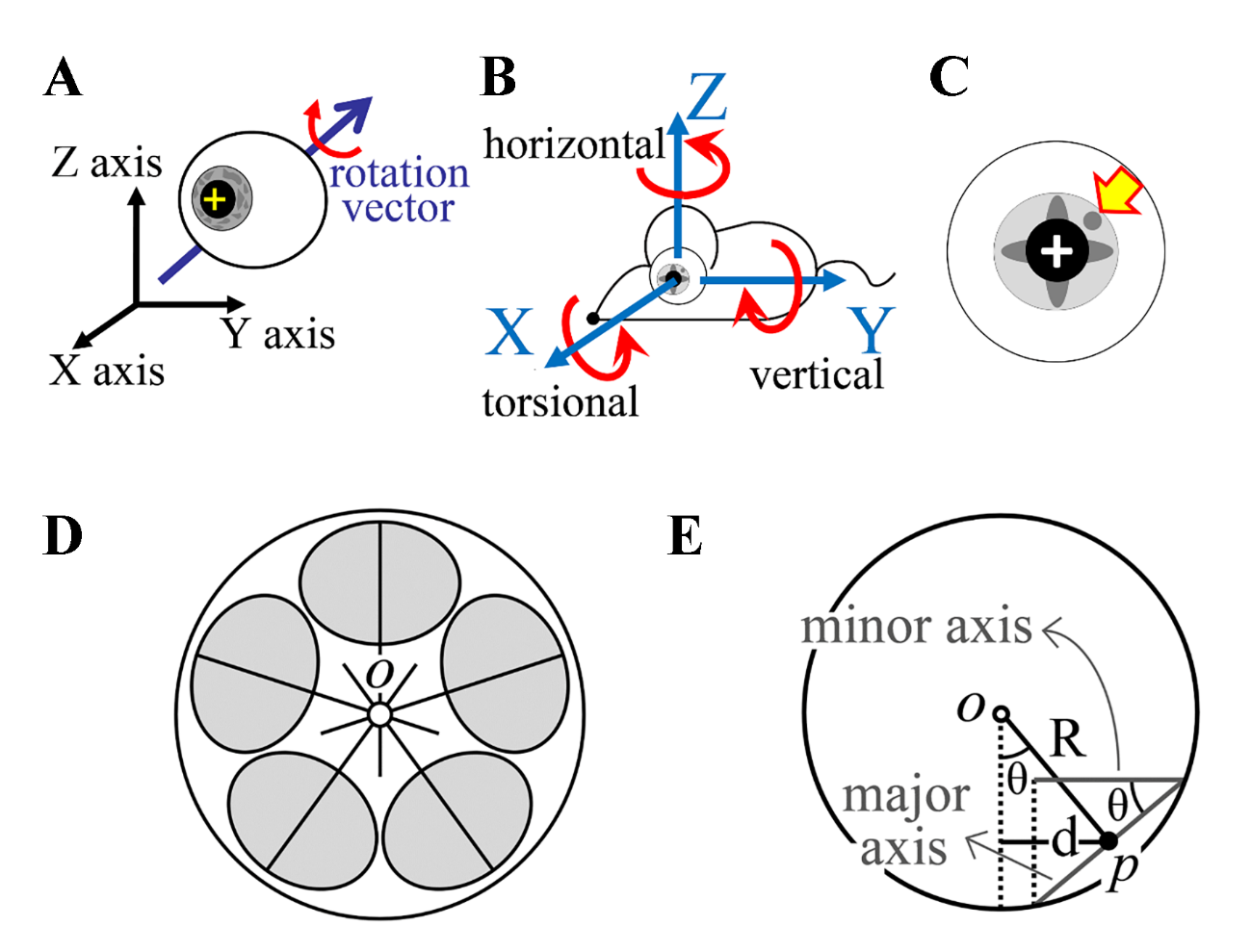


**Supplementary Figure 1.** Method to analyze the three-dimensional eye position in mice. (A) Rotation vector of eye position. Any eye position can be attained by rotating the eye from the reference position along a single axis. Therefore, any eye position can be represented by the axis and rotation angle around the axis. A rotation vector is a vector whose direction is the axis, and its magnitude is the value of tan (rotation angle/2). (B) Three-dimensional coordinate frame of the left eye. The three-dimensional coordinates of the left eye were as follows: X axis parallel to the interaural axis (positive left), Y axis parallel to the naso-occipital axis (positive backward), and Z axis normal to the X–Y plane (positive upward). In this study, eye movements were described three-dimensionally using the axis angle, characterizing the eye positions around a single rotation. Therefore, X, Y, and Z components mainly reflect the roll, pitch, and yaw components, respectively. (C) Schematic of the mouse eye image. The coordinates at the center of the pupil and iris freckles, indicated by arrows, were extracted. (D) Method to determine the center of eye rotation coordinates. The edge of the pupil was detected and approximated using an ellipse. Subsequently, the minor and major axes of the ellipse were determined. The center of eye rotation (o) on the image plane was determined as the intersection of the extensions of the minor axes. (E) Method to calculate the radius of rotation of the pupil center.

After determining the center of eye rotation, we calculated the radius of rotation of the center of the pupil (R) using the following formula:

R√(1-((length of minor axis)⁄(length of major axis))^2 )=d

Here, *d* is the distance between the center of the eye rotation *o* and the center of the pupil ellipse *p*.


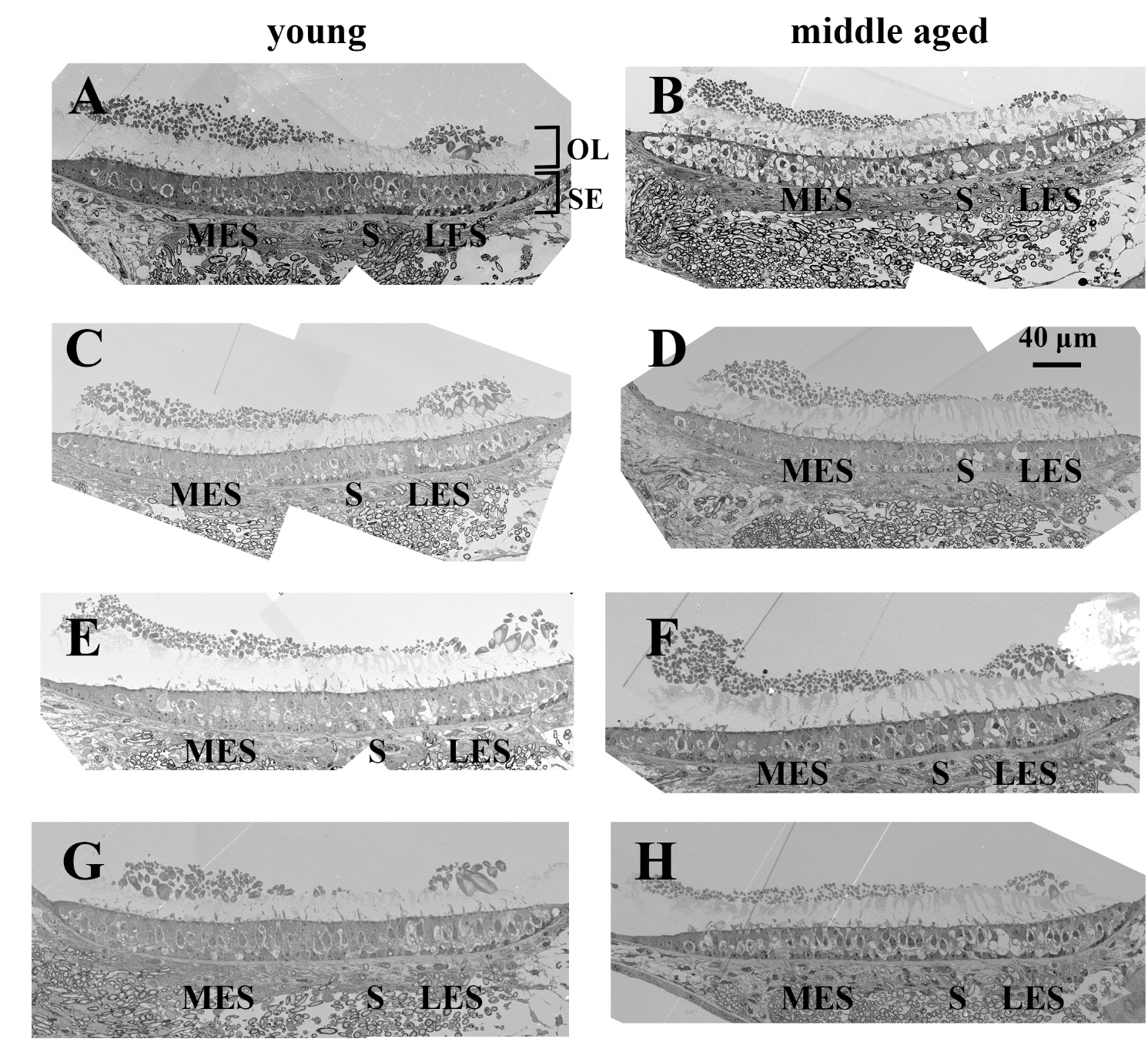


**Supplementary Figure 2.** Electron microscopy images of the striola in young (**A**, **C**, **E**, and **G**) and middle-aged (**B**, **D**, **F**, and **H**) mice.


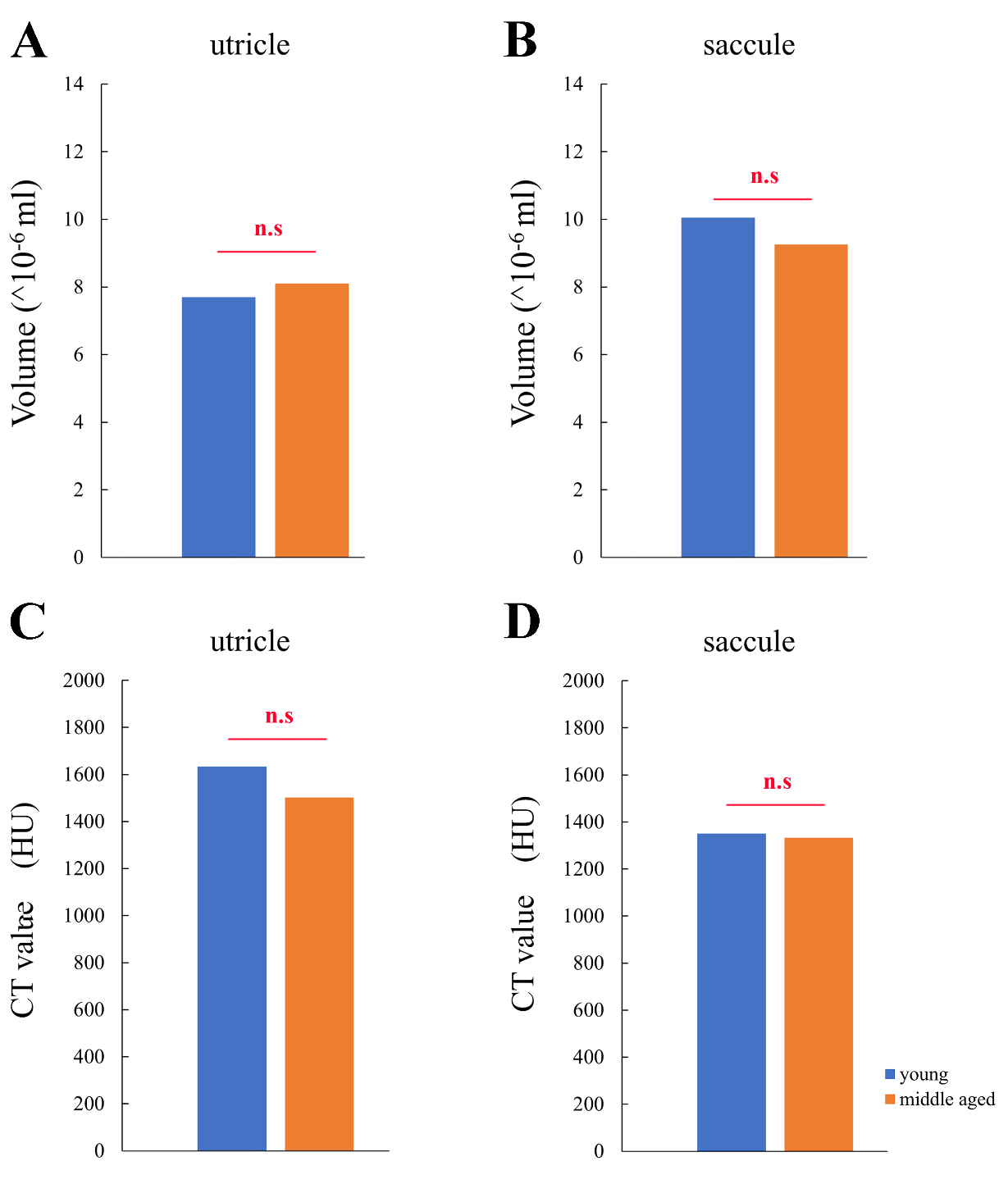


**Supplementary Figure 3.** Comparison of the μCT images of young and middle-aged female mice. (**A**) No significant differences were observed in the utricle volume (P=0.70, Mann–Whitney *U* test). (**B**) No significant differences were observed in the saccule volume (P=0.46, Mann–Whitney *U* test). (**C**) No significant differences were observed in the utricle CT number (P=0.054, Mann–Whitney *U* test). (**D**) No significant differences were observed in the saccule CT number (P=0.90, Mann–Whitney *U* test).


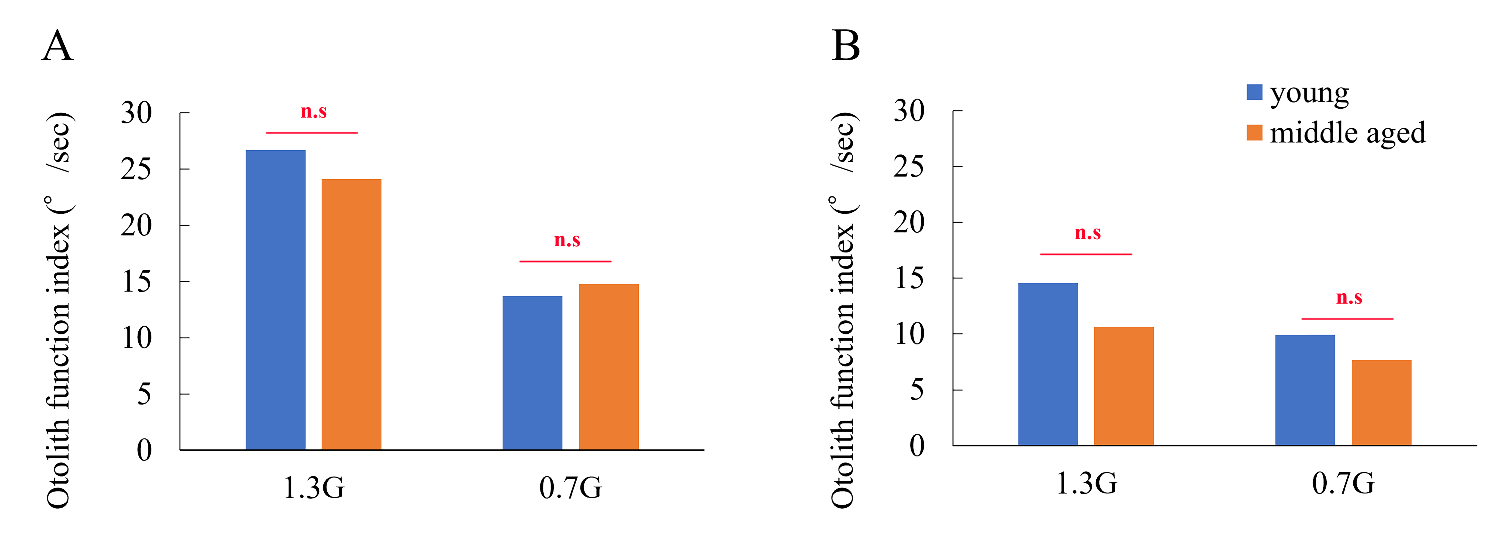


**Supplementary Figure 4.** Comparison of the linear vestibulo-ocular reflex (LVOR) results of young and middle-aged female mice. (**A**) Otolith function index during lateral acceleration. No significant differences were observed at both accelerations (1.3G [P=0.73] and 0.7G [P=0.73]). (**B**) Otolith function index during longitudinal acceleration. No significant differences were observed at both accelerations (1.3G [P=0.19] and 0.7G [P=0.24]).
